# A haplotype-resolved genome assembly of *Malus domestica* ‘Red Fuji’

**DOI:** 10.1038/s41597-024-03401-1

**Published:** 2024-06-06

**Authors:** Haixu Peng, Yating Yi, Jinrong Li, You Qing, Xuyang Zhai, Yulin Deng, Ji Tian, Jie Zhang, Yujing Hu, Xiaoxiao Qin, Yanfen Lu, Yuncong Yao, Sen Wang, Yi Zheng

**Affiliations:** 1 Beijing Key Laboratory for Agriculture Application and New Technique, Colege of Plant Science and Technology, Bejing University of Agriculture, Bejing, 102206 China; 2 Bioinformatics Center, Bejing University of Agriculture, Bejing, 102206 China

**Keywords:** Plant genetics, Genome informatics

## Abstract

The ‘Red Fuji’ apple (*Malus domestica*), is one of the most important and popular economic crops worldwide in the fruit industry. Using PacBio HiFi long reads and Hi-C reads, we assembled a high-quality haplotype-resolved genome of ‘Red Fuji’, with sizes of 668.7 and 668.8 Mb, and N50 sizes of 34.1 and 31.4 Mb. About 97.2% of sequences were anchored in 34 chromosomes. We annotated both haploid genomes, identifying a total of 95,439 protein-coding genes in the two haplotype genomes, with 98% functional annotation. The haplotype-resolved genome of ‘Red Fuji’ apple stands as a precise benchmark for an array of analyses, such as comparative genomics, transcriptomics, and allelic expression studies. This comprehensive resource is paramount in unraveling variations in allelic expression, advancing quality improvements, and refining breeding efforts.

## Background & Summary

The apple (*Malus domestica*), belonging to the *Rosaceae* family and *Malus* genus, is primarily cultivated in temperate regions. As an economically and nutritionally important fruit crop worldwide, the world’s apple production reached an annual production of approximately 93.14 million tons, of which China represented nearly half of the global annual production (45.98 million tons, https://www.fao.org). Among various cultivated varieties, the ‘Red Fuji’ apple has become a globally popular variety, especially in China, due to its long-term storage capability and high-quality fruit characteristics^1^. Previous studies had demonstrated several genes related to the long-term storage of ‘Fuji’, like *MdPG1* and *MdACS1*^2^. Additionally, genes like *MdbHLH3* and *MdMYB10* enhance ethylene production and anthocyanin accumulation, thus significantly influencing fruit quality^3–6^. Despite the genome of various apple varieties being published^7–9^, the haplotype-resolved genome of ‘Red Fuji’ remains unexplored.

“Red Fuji” apple is a bud mutation variant derived from the “Fuji” variety, which itself is a hybrid of ‘Rall Janet’ and ‘Delicious’, maintaining excellent traits of both parents, including good storage resistance and a crisp, sweet flavor^10^. Constructing a haplotype-resolved genome contributed to identify genome variations between homologous chromosomes including Single Nucleotide Polymorphism (SNP), Structure Variation (SV), and explore issues like allele-specific gene expression, furthermore elucidate biological traits of its parents (e.g., storage period, flavor) by comparative genomic analysis^8,11–13^. Therefore, decoding the haplotype-resolved genome of ‘Red Fuji’ plays a vital role in uncovering unique parental traits and understanding genetic disparities among individuals.

**Fig. 1:**
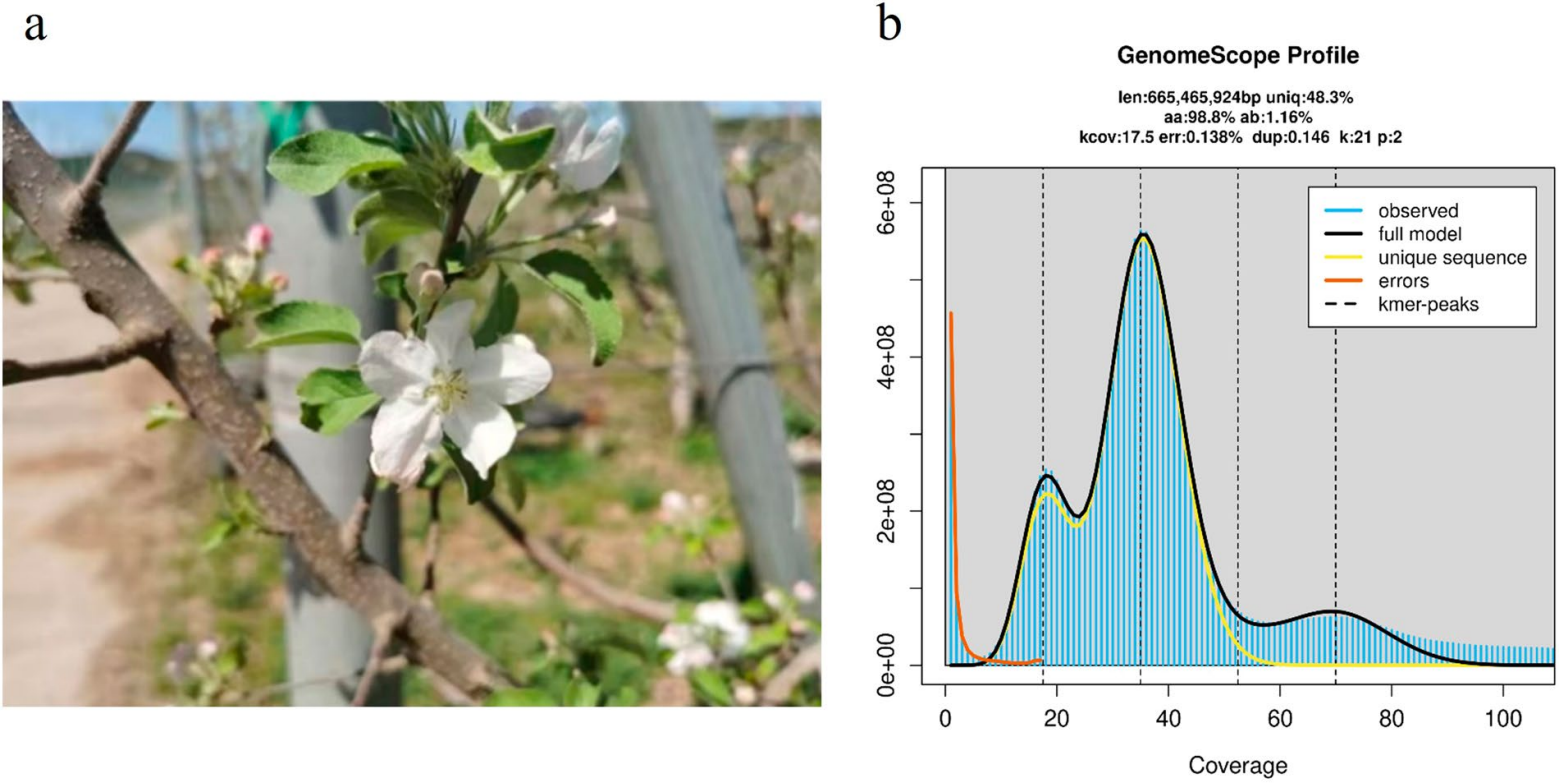
(**a**) A photograph of the ‘Red Fuji’ apple tree in the feld. (**b**) Kmer-21 histogram generated using PacBio HiFi reads. Genome size and heterozygosity rate were estimated using GenomeScope2.

By employing an integrative approach, we combined PacBio HiFi and Hi-C data to conduct *de novo* assembly. We obtained approximately 24 Gb of high-quality PacBio HiFi reads with an average length of 16,371 bp, the genome size is approximately 665.4 Mb, used for calculating the sequencing depth. (Table 1). Based on k-mer analysis, the estimated genome size was 665.4 Mb (Fig. 1b), suggesting a sequencing depth of approximately 39.4 × . We obtained 415 and 230 contigs of two haplotypes, with genome sizes of 668.76 Mb and 668.82 Mb, respectively. The N50 values were 34.09 Mb and 31.49 Mb (Table 2), respectively. We designated the two haploid genomes as hapA (A) and hapB (B). Among them, the A genome and B genome each had 17 contigs anchored to the 17 chromosomes of the ‘Red Fuji’ apple. The final assembled genome sizes for the ‘Red Fuji’ apple were 648.33 Mb for the A genome and 651.97 Mb for the B genome, with anchored rates of 96.90% and 97.40%, respectively. The analysis revealed that the total size of the genome is approximately 1.3 Gb, with a total of 861.83 Mb of sequence identified as repetitive elements, accounting for approximately 64.43% of the entire genome. Additionally, we conducted gene annotation on the two haploid genomes, identifying 47,655 and 47,784 genes for A and B genome, respectively (Fig. 3, Table 3). The assessment of genome completeness and continuity using BUSCO showed highly quality results, with completeness scores of 98.9% and 99.0% for the two haploid genomes (Table 2), respectively.

**Table 1:** Statistics of genomic sequencing data.

	PacBio-sequel II data	Illumina paired-end data
Number of Reads	1,507,866	544,939,732
Number of Bases (bp)	24,685,326,241	81,740,959,800
Coverage	40	136
Mean (bp)	16,371	150
Minimum (bp)	84	150
Maximum (bp)	54,554	150

**Table 2:** Statistics of the *Malus domestica* cv. ‘Red Fuji’ haplotype genomes.

	Chromosomes	Chromosomes
Sequence#	17	17
Sequence (bp)	648,338,598	651,978,723
Shortest (bp)	30,346,802	31,378,059
Longest (bp)	48,082,340	48,074,198
Average (bp)	38,137,565	38,351,690
N50 (bp)	38,559,328	38,258,856
L50	8	8
N90 (bp)	31,774,720	31,653,163
L90	15	15
GC content (%)	38.08	38.09
Complete BUSCOs (%)	98.90	99.00
Complete and single-copy BUSCOs (%)	62.10	61.80
Complete and duplicated BUSCOs (%)	36.80	37.20
Mapping ratio(PacBio%)	99.93	99.93
Mapping ratio(Illumina%)	99.82	98.27

**Fig. 2:**
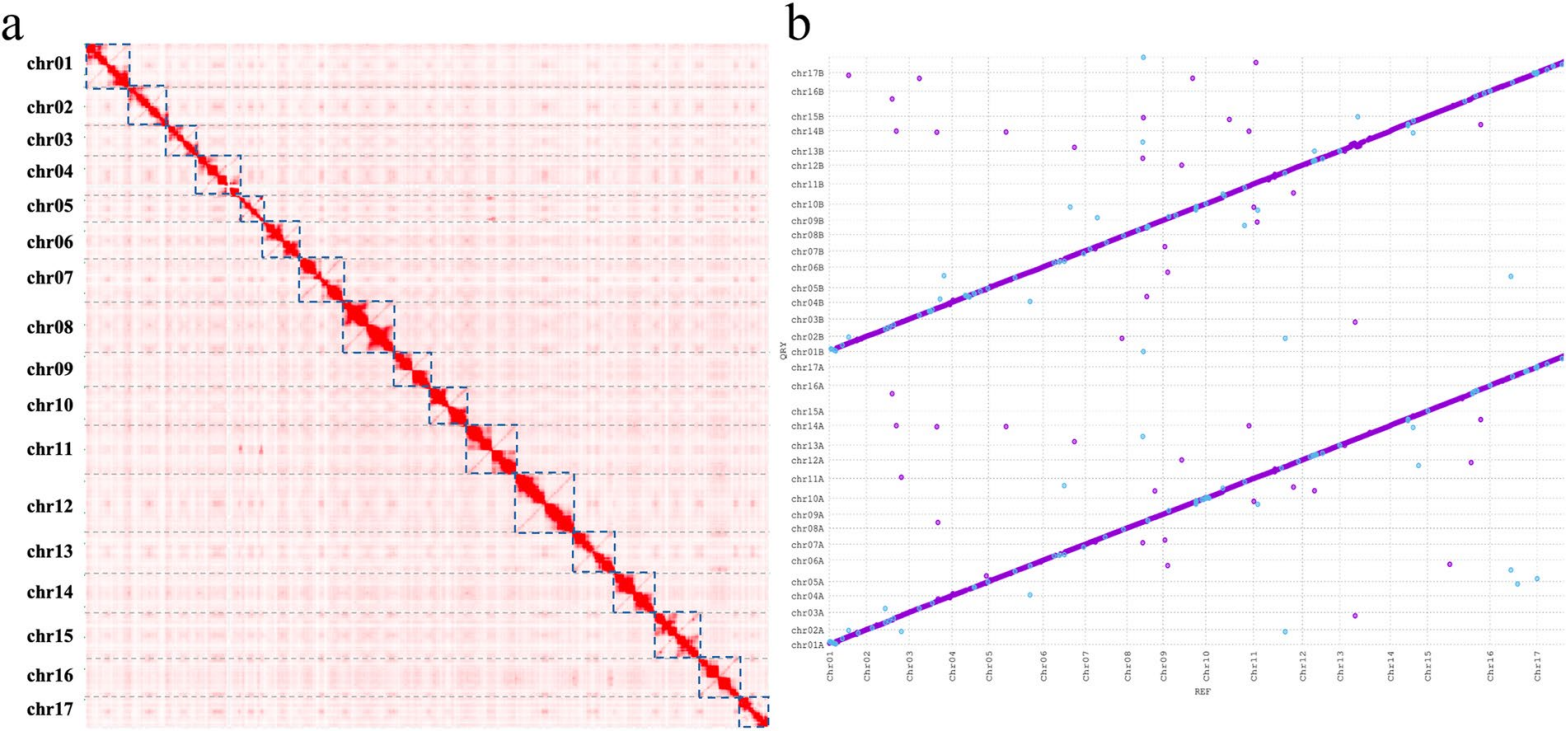
(**a**) The Hi-C heatmap of chromosome interactions in *M. domestica* chromosomes. (**b**) Sequence collinearity between the two sets of ‘Red fuji’ haplotype genomes and ‘GDDH13’.

**Fig. 3:**
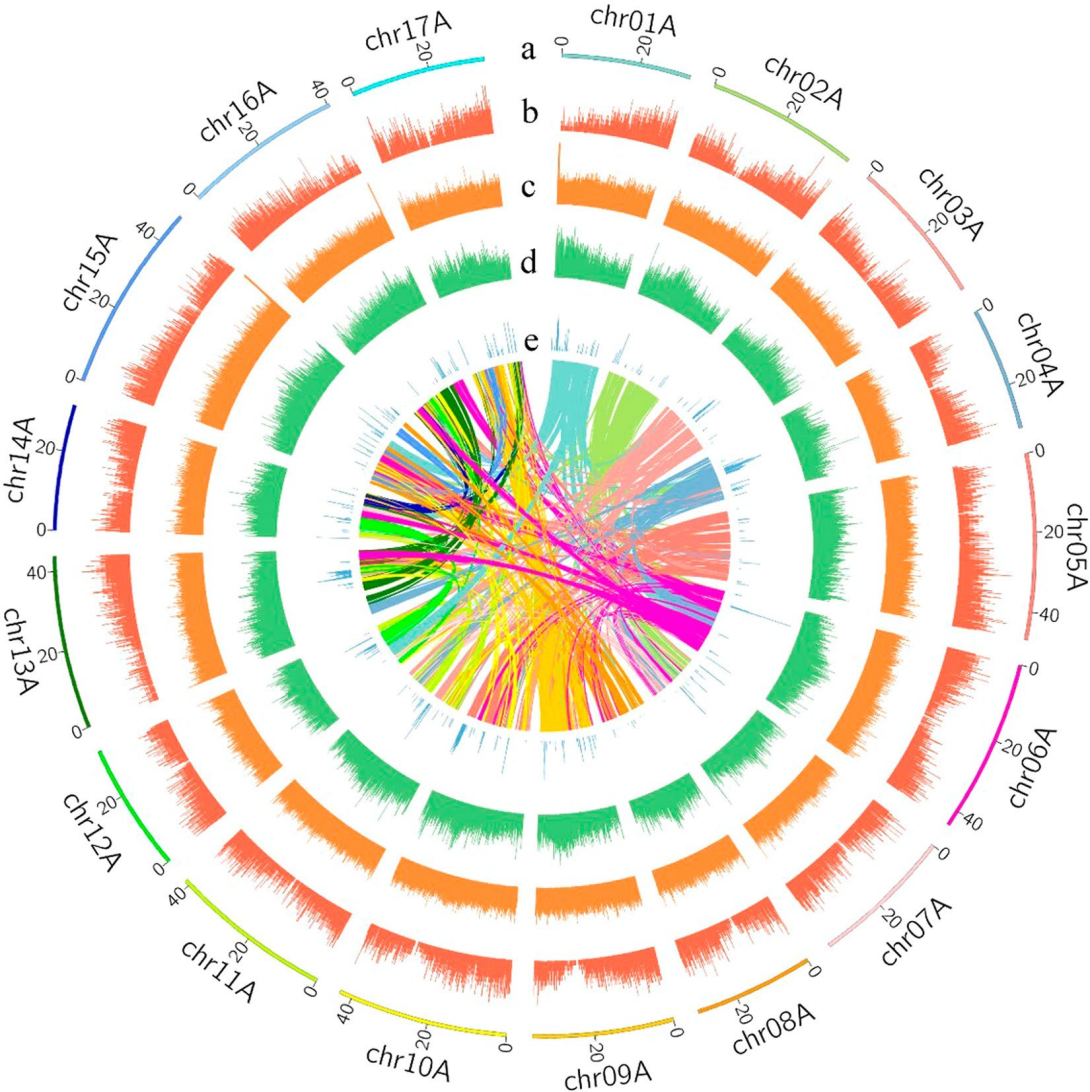
Circular map of *M. domestica*. (**a**) Chromosome ID and length, (**b**) Density of protein-coding genes, (**c**) Density of LTR elements, (**d**) GC content, (**e**) Density of indel, (**f**) Paralog synteny relationships.

**Table 3:** Statistics of gene models of the *Malus domestica* cv. ‘Red Fuji’ haplotype genomes.

	HapA	HapB
Gene number	47,655	47,784
Gene density (gene/Mb)	65	66
Mean gene size (bp)	3,361	3,390
Mean CDS size (bp)	1,285	1,288
Mean exon number per gene	5.4	5.4
Mean exon size (bp)	212	238.68
Mean intron number per gene	4.4	4.4
Mean intron size (bp)	489	494
Coding GC contnet (%)	42.84	42.85
Intron GC content (%)	32.58	32.57

## Methods

### Materials

In April 2021, young and tender leaves were collected from ‘Red Fuji’ apple trees at the Foss Apple Orchard in Miyun District, Beijing, five days after flowering (Fig. 1a). The collected leaves were immediately frozen in liquid nitrogen for PacBio HiFi and Hi-C library preparation and sequencing. The related sequencing services were performed by Berry Genomics Co. Ltd.(Beijing, China).

### DNA extract

The genomic DNA (gDNA) extraction from young and tender leaf samples of the ‘Red Fuji’ apple was performed using the CTAB method. The extracted DNA underwent quantification and quality assessment using the NanoDrop 2000 spectrophotometer (Thermo Fisher Scientific), agarose gel electrophoresis, and a Qubit fluorometer (Invitrogen). Following the analysis, DNA purification was conducted using AMPure PB beads (PacBio 100-265-900) to obtain high-quality gDNA with a concentration of ≥100 ng/μl and a yield of ≥10 μg for subsequent library construction.

### HiFi SMRTbell Library Construction and Sequencing

The PacBio SMRTbell (Single-molecule real-time) library was constructed using the SMRTbell® Express Template Prep Kit 2.0 (Pacific Biosciences, PN 101-853-100). The specific procedures and checklist for preparing HiFi SMRTbell Libraries using the SMRTbell Express Template Prep Kit 2.0 can be found at this link: Procedure Checklist - Preparing HiFi SMRTbell Libraries using SMRTbell Express Template Prep Kit 2.0. In summary, the library construction involves the following steps: Pulsed-field gel electrophoresis is performed to assess the 10 μg gDNA for library construction. The majority of DNA fragments must exceed 40 Kb to meet the library construction requirements. Once the DNA is qualified, the fragments are sheared to 15 Kb using the Megaruptor instrument (Diagenode B06010001). The DNA is then concentrated using AMPure® PB Beads (Pacific Biosciences 100-265-900). Subsequently, the SMRTbell library is constructed using the 2.0 kit, which includes steps such as removal of single-stranded overhangs, DNA damage repair, end repair, A-tailing, adapter ligation, and enzyme digestion. Fragment screening is performed using either the SageELF system (Sage Science ELF000) or the BluePippin system (Sage Science BLU0001). The qualified libraries are evenly distributed onto SMRT Cells and subjected to 30 hours of sequencing using the Sequel II system (Pacific Biosciences, CA, USA).

### Hi-C Library Construction and Sequencing

The Hi-C library construction and sequencing workflow involve several steps, including formaldehyde treatment, restriction enzyme digestion, biotin labeling, ligation with connector enzymes, DNA capture with labeled probes, library construction, and sequencing. The specific process for Hi-C library construction and sequencing is as follows: Cells are treated with a cell cross-linking agent, formaldehyde, to establish DNA-protein crosslinks, fixing the conformation of DNA. The cells are lysed, and high-quality DNA is extracted. The crosslinked DNA is then subjected to restriction enzyme digestion. End-repair is performed, and biotin is introduced to label the oligonucleotide ends. Connector enzymes are used to ligate the neighboring DNA fragments together. Protein digestion is carried out at the ligation junctions to release the protein-DNA crosslinks, dissociating the protein from the DNA. The DNA is purified and recovered, enzymatically fragmented, labeled DNA is captured, sequencing adapters are added, and the sequencing library is constructed. The PE150 sequencing mode is performed using the Illumina NovaSeq 6000 sequencing platform.

### Genome size and ploidy analysis

We employed the k-mer analysis method to estimate the heterozygosity, ploidy, and genome size of the ‘Red Fuji’ apple. The 24 Gb of high-quality PacBio HiFi reads obtained from the Sequel II system will be utilized for genome size and ploidy analysis. Firstly, the reads were subjected to k-mer counting (k = 21) using the Jellyfish^14^ software. The Genomescope^15^ software was then employed to analyze the k-mer counting results and calculate the genome size and heterozygosity of the ‘Red Fuji’ apple, as well as predict its ploidy. Following analysis, the genome size is estimated to be approximately 665.4 Mb with 1.18% heterozygosity, and it has been identified as a diploid genome (Fig. 1b).

### Genome assembly

The initial assembly of the ‘Red Fuji’ apple genome was performed using the “Hi-C integrated” mode of Hifiasm^16^. In this mode, default parameters (‘--hom-cov 34 --n-weight 6 -s 0.45 -O 2 –h1 Fuji_hic.R1.fastq.gz --h2 Fuji_hic.R2.fastq.gz’) were used, and both PacBio HiFi reads and Hi-C paired-end sequencing data were provided for the preliminary assembly. This approach yielded two haplotype contig sequences. The preliminary assembly sequences were converted to the FASTA format for further analysis.

Using Hi-C interaction data, the contigs were scaffolded using the Juicer^17^ and 3D-DNA^18^ pipeline. Firstly, utilizing Juicer, the prediction of restriction enzyme sites in the genome was conducted, while simultaneously aligning the Hi-C data to the genome. The automated error correction, contig ordering, orientation, and scaffolding were accomplished by 3D-DNA (parameters: -m haploid -r 0). The results generated in the previous step were manually refined using JuiceBox^17^, which included correcting chromosome boundaries, rejoining misjoins, and addressing inversions and translocations (Fig. 2a). Contigs representing each chromosome were recorded in an AGP format file, and the final genome was generated using agp2fa mode of RagTag^19^.

### Genome annotation

Supported by three sources of evidence, repeat sequence annotation involved *de novo* prediction of internal repeat sequences, as well as utilizing the public databases Dfam and RepBase. Internal repeat sequences were identified using RepeatModeler^20^. The identified repeat sequences, integrated with Dfam and RepBase, were then used by RepeatMasker^21^ to mask repeat sequences within the genome. Additionally, the TRF^22^ software was employed to predict the tandem repetitive sequences.

Protein-coding genes in the ‘Red Fuji’ genome were predicted by three methods: *ab initio* gene prediction, homology-based gene prediction, and transcript-based gene prediction. For transcript-based evidence involved using HISAT2^23^ and StringTie^24^ for genome-wide assembly, the assembled transcripts were subjected to ORF region prediction using TransDecoder (https://github.com/TransDecoder/TransDecoder) and served as transcript-based evidence. The homology-based prediction used protein sequences derived from five different apple varieties: *Malus sieversii* (https://www.rosaceae.org/Analysis/10816132), *Malus sylvestris* (https://www.rosaceae.org/Analysis/10816134), *Malus domestica* cv. Golden Delicious (https://www.rosaceae.org/species/malus/malus_x_domestica/genome_GDDH13_v1.1), *M. domestica* cv. Gala (https://www.rosaceae.org/Analysis/10816131) and *M. domestica* cv. HFTH1 (https://www.rosaceae.org/species/malus_x_domestica_HFTH1/genome_v1.0) were utilized to perform homology prediction by Exonerate^25^. Before *ab initio* gene prediction, the genome sequences were hard masked by RepeatMasker. The assembled transcripts were used as input filesto generate training models using Augustus^26^ and BRAKER2^27^, and a total of 90,470 coding genes were ultimately predicted in the *de novo* analysis. Finally, the Maker software^28^ was used to integrate the evidence generated by the aforementioned methods, resulting in the final set of protein-coding genes. The completeness and continuity of the annotated protein-coding genes were assessed using BUSCO^29^, the results indicate that hapA and hapB harbor 1586 (98.3%) and 1587 (98.3%) complete BUSCOs for protein-coding genes, respectively.

### Functional annotation of the genome

For the functional annotation of the protein-coding genes, we employed a standardized workflow^30,31^. In summary, the protein sequences of the two haplotype genomes were compared against several protein databases using the DIAMOND program, with an E-value cutoff of 1E-4. The protein databases included the non-redundant protein database (NR), TrEMBL (http://www.uniprot.org/), SwissProt^32^, and the Arabidopsis protein database. The BLAST results from SwissProt, TrEMBL, and the Arabidopsis protein database were loaded into the AHRD (https://github.com/asishallab/AHRD) program to predict concise, informative, and accurate functional descriptions for each gene.

Furthermore, the protein sequences were compared against the InterPro database^33^ using InterProScan to identify functional protein domains. The BLAST results from the NR database were combined with the identified InterPro protein domains to perform Gene Ontology (GO) annotation using the Blast2GO program^34^, and the GO terms were associated with the annotated protein sequences.

To explore the protein-coding genes’ connections to pathways, the ‘Red Fuji’ apple protein sequences were annotated using the online KAAS tool (https://www.genome.jp/tools/kaas/). This annotation provided information about the genes’ associations with various metabolic pathways. Additionally, the iTAK software was used to predict transcription factors, transcription regulators, and protein kinases.

The protein-coding genes were compared separately with the NR, SwissProt, Arabidopsis protein database, and TrEMBL databases. Each database annotated 95,552, 77,453, 67,243, and 92,132 genes, respectively. A total of 52,073 genes were matched to 2,555 GO terms. Additionally, in the pathway annotation, 32,312 genes were annotated to 143 ko IDs. In the prediction of transcription factors (TFs) and transcription regulators (TRs), a total of 6,283 genes belong to 93 different gene families. Additionally, 3,119 genes were annotated to 127 different protein kinase (PK) families. By comparing with the Pfam database, a total of 4,191 protein functional domains were identified, and distributed among 61,043 different genes (Table 4).

**Table 4:** Functional annotation of genes in the *Malus domestica* cv. ‘Red Fuji’ haplotype genomes.

	HapA		HapB	
	Gene number	Percent (%)	Gene number	Percent (%)
NR	47,707	99.64	47,845	99.89
*A.thaliana*	33,591	70.16	33,652	70.25
SwissProt	38,676	80.78	38,777	80.95
TrEMBL	46,006	96.09	46,126	96.30
AHRD	43,339	90.84	43,464	90.85
GO	25,991	54.29	26,082	54.45
KEGG	16,228	33.90	16,084	33.58
TF/TR	3,135	6.55	3,148	6.57
PK	1,560	3.26	1,559	3.25
Pfam	30,523	63.75	30,526	63.73

### Genome collinearity analysis

**Fig. 4:**
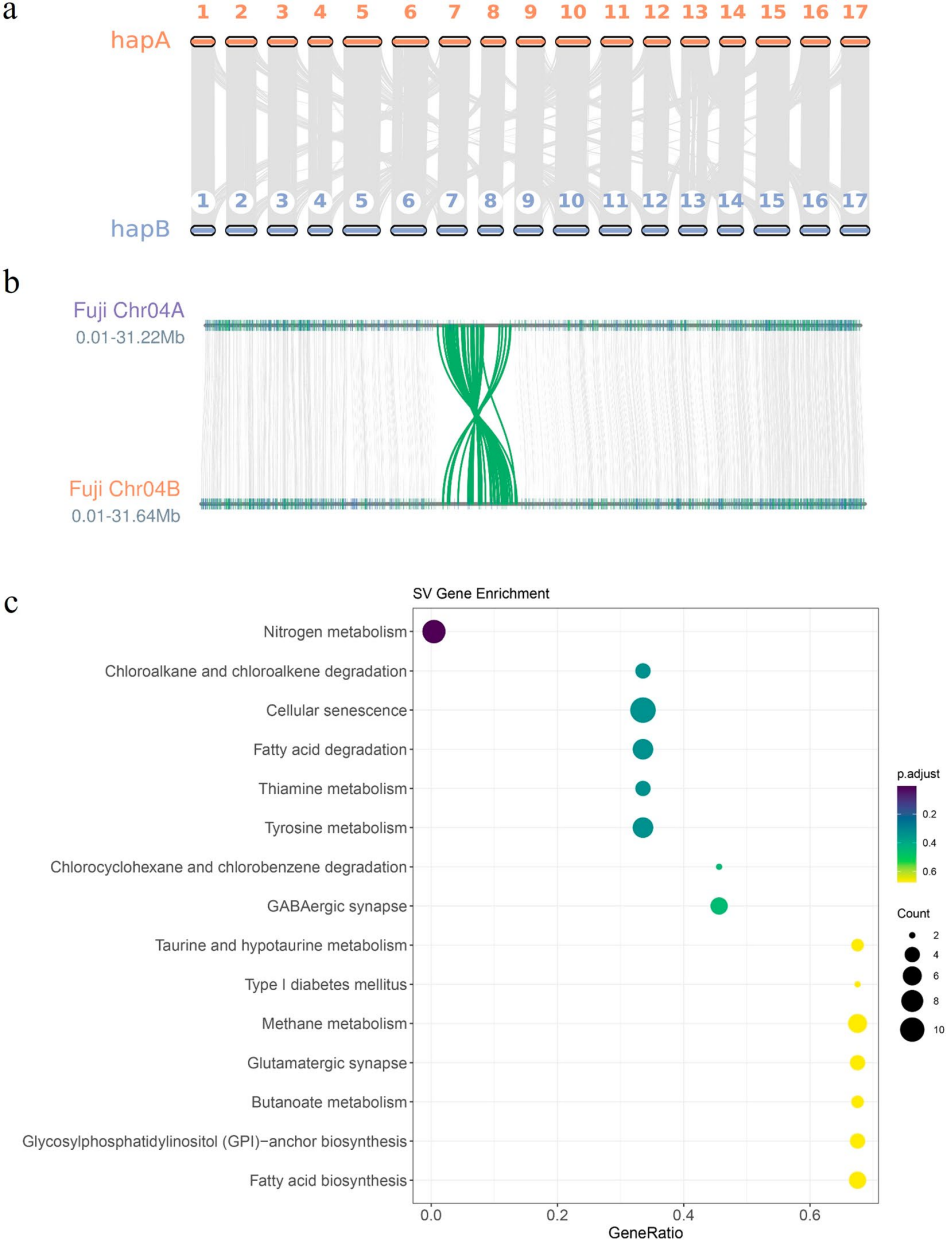
(**a**) Genome synteny between the two haplotype genome (**b**) An inversion region about 1.5 Mb long on chromosome 4. (**c**) KEGG enrichment of SV-related genes.

The homologous proteins between the two haplotype genomes of ‘Red Fuji’ were identified using DIAMOND^35^. MCScanX^36^ was then employed to assess the synteny between the haplotype genomes, requiring a minimum of five syntenic genes and no more than 15 gapped genes. A total of 1216 collinear blocks were identified, involving 38,701 pairs of genes, which accounts for 81.38% of the total gene count. The genome synteny analysis indicated a stronger degree of synteny between the two haplotype genomes of ‘Red Fuji’ (Fig. 4a).

### Structural variation detection

Detect intra-species structural variations (SVs) between the two haplotype-resolved genomes with the SyRI pipeline. Firstly, the haplotype B genome was aligned to the haplotype A genome using Mummer^37^ with parameters set as “--maxmatch -c 100 -l 500”. The raw alignments results were further filtered using delta-filtre with parameters: -m -i 90 -l 100. Additionally, the two haplotype genomes were aligned by minimap2^38^, with parameters set as “--eqx -x asm5 -c”. The resulting delta and PAF files were predicted structural variations using SyRI pipeline^39^ with default parameters.

Structural variations associated with genes were defined as those overlapping with genes and their upstream regions within 3 Kb. Detected variations from SyRI included two levels of structural changes: genomic rearrangements and sequence variations, the latter occurring in rearranged and collinear regions. The variations in rearranged and collinear regions are used for subsequent SV analysis, while embedded rearrangements are not included in further analysis. After applying the aforementioned method, we identified seven types of structural variation sites. Specifically, there were 2,922 insertions and 3,040 deletions, along with 373 inversions. Finally, 5,028 genes were found to be associated with the detected structural variations. Furthermore, a 1.2 Mb inversion region was identified on chr04 (Fig. 4b), subsequent analysis revealed their significant influence on nitrogen metabolism and fatty acid metabolism in the ‘Red Fuji’ apple (Table S1, Fig. 4c).

## Data Records

The PacBio HiFi and Hi-C sequencing data that were used for the genome assembly have been deposited in the NCBI Sequence Read Archive with accession number SRR24517737 and SRR24517738^40^, under BioProject number PRJNA971677. The RNA-seq data used for the genome annotation were deposited in the NCBI, under BioProject number PRJNA555185. The chromosomal assembly and dataset of gene annotation have been deposited in Figshare^41^ (10.6084/m9.figshare.23803938). The assembled diploid genome of ‘Fuji’ was deposited in GenBank database^42,43^ (accession number: GCA_037303155.1 for hapA and GCA_037312365.1 for hapB).

## Technical Validation

We primarily assessed genome completeness from two perspectives, the assembled genome sequences and the annotated protein sequences. For the assembled genome sequences, we employed the following five methods:

We conducted a comparative analysis of the assembled sequence against three published apple genomes (*Malus domestica* cv. Golden Delicious, *M. domestica* cv. HFTH1, *M. domestica* cv. Gala) using the MUMMER software (version 4.0.c)^37^ to assess collinearity (Fig. 2b). We used BUSCO(v5.2.1) to compare the genome sequence against the embryophyta_odb10 core gene database, as ‘Red Fuji’ apple belongs to the angiosperms. This comparison allowed us to evaluate the completeness of the genome. We aligned the quality-controlled PacBio HiFi sequencing data to the assembled ‘Red Fuji’ apple genome using Minimap2^38^. The mapping rate was calculated by a python script, and its was 99.93% for both haploid genomes (Table 2). We also calculated assembly metrics such as contig/scaffold N50 and L50 to assess the quality of the assembled genome. We mapped transcriptome data obtained from Illumina sequencing platforms to the two haploid assembled genomes using the HISAT2 software.

The primary contigs of the two haplotypes from the hifiasm assembly result were evaluated using the BUSCO pipeline based on the embryophyta_odb10 database (Table 2). Based on the database alignment, it was found that the two haploid genomes consist of 1596 and 1597 homologous genes, respectively. This observation indicates that both haploid genomes boast an impressive completeness, surpassing 98.9%. These results affirm the high quality and integrity of the assembled genomes.

### Supplementary information

Supplementary Information

## Data Availability

All software and pipelines were executed according to the manual and protocols of the published bioinformatics tools. The version and code/parameters of software have been described in Methods.
